# Comparison of ipsilateral breast tumor recurrence after breast-conserving surgery between ductal carcinoma in situ and invasive breast cancer

**DOI:** 10.1186/s12957-016-0885-6

**Published:** 2016-04-27

**Authors:** Young Jin Choi, Young Duck Shin, Young Jin Song

**Affiliations:** Department of Surgery, Chungbuk National University School of Medicine, 410 Sungbong-ro, Heungdeok-gu, Cheongju, 361-763 South Korea; Department of Anesthesiology, Chungbuk National University School of Medicine, 410 Sungbong-ro, Heungdeok-gu, Cheongju, 361-763 South Korea

**Keywords:** Ipsilateral breast tumor recurrence (IBTR), Breast-conserving surgery (BCS), Ductal carcinoma in situ (DCIS), Breast carcinoma, Margin, Radiotherapy, Survival

## Abstract

**Background:**

We aimed to evaluate the differences in the rates and predictive factors for ipsilateral breast tumor recurrence (IBTR) after breast-conserving surgery (BCS) between ductal carcinoma in situ (DCIS) and invasive breast cancer. And, we evaluated the impact of IBTR on overall survival and distant metastasis.

**Methods:**

We retrospectively reviewed 322 consecutive patients with DCIS or invasive breast cancer who underwent BCS between 2004 and 2010. We evaluated the rates of IBTR of DCIS and invasive breast cancer. Univariate and multivariate analyses were performed to determine the predictive factors for IBTR, and survival rates were analyzed with Kaplan-Meier estimates.

**Results:**

With a median follow-up period of 57 months, 5 (10 %) out of 50 DCIS patients and 14 (5.1 %) out of 272 invasive cancer patients had developed IBTR. Factors associated with IBTR on univariate and multivariate analyses were positive resection margin status in DCIS and omission of radiotherapy in invasive cancer, respectively. The hormone receptor negativity was strong independent predictive factors for IBTR in both DCIS and invasive breast cancer. Although the differences of survival curve did not reach statistical significance, the 5-year overall survival and distant metastasis-free survival of invasive cancer patients who suffered IBTR were inferior to those without (84 vs. 98 % and 63.3 vs. 96.5 %, respectively). Advanced initial stage, lymph node metastasis and experience of IBTR were associated with poor overall survival and distant metastasis on univariate and multivariate analyses.

**Conclusions:**

The hormone receptor negativity was revealed as independent predictive factor for IBTR after BCS in both DCIS and invasive cancer. Experience of IBTR was independent prognostic factor for poor overall outcome in patients with invasive breast cancer. Aggressive local control and adjuvant therapy should be made in hormone receptor-negative patients who receive treatment with BCS.

## Background

Breast cancer is the second most common newly diagnosed malignancy in Korean women. The number of early breast cancer detections has increased due to the development of screening programs, and the rates of breast-conserving surgery (BCS) have also increased. The rate of BCS was as high as 62 % at 2012, according to the nationwide database of the Korean Breast Cancer Society.

Breast-conserving treatment, defined as lumpectomy or quadrantectomy followed by radiation therapy, is the standard surgical method in the treatment of invasive breast cancer. The results of 20 years of follow-up from the National Surgical Adjuvant Breast and Bowel Project (NSABP) B-06 trial showed no significant differences in disease-free survival, distant disease-free survival, or overall survival between the BCS and mastectomy groups [1]. However, the incidence of ipsilateral breast tumor recurrence (IBTR) after BCS is 5–10 % during 5 years of follow-up, and the risk continues over a longer period of follow-up [1, 2]. Positive margin status, human epidermal growth factor receptor 2 (HER-2) positivity, young age, in situ lesions around the tumor, triple-negative subtype, and lymphovascular invasion (LVI) of tumors are known as predictive factors for the development of IBTR after BCS [3–7]. Nevertheless, the rationale for performing BCS is that the overall survival (OS) was not different compared to the mastectomy group.

BCS has also been the standard surgical method in patients with ductal carcinoma in situ (DCIS). The 5-year rate of IBTR after BCS in DCIS has been reported to be about 5–10 %. Young age, positive or close resection margins, and omission of endocrine therapy and radiotherapy have been associated with an increased risk of IBTR after BCS in DCIS patients [8–10]. Negative surgical margins should be obtained for patients with DCIS after BCS regardless of radiotherapy. Numerous studies have found that margin thresholds greater than 10 mm are warranted for the treatment of patients with DCIS who undergo BCS [9, 11]. There is a difference in the appropriate margin threshold between DCIS and invasive cancer, and 2014 American Society of Clinical Oncology (ASCO) guidelines recommend the use of “no ink on tumor” as the standard for an adequate margin in invasive cancer [12].

There have been many controversies regarding the effect of IBTR on distant recurrence and survival. Many investigators have reported that IBTR after BCS is associated with subsequent mortality. Studies showed that early IBTR within 2 years after BCS was associated with a higher risk of distant relapse and mortality [13–15]. Clinicopathologic features of the recurrent tumor including LVI, high-grade histology, high Ki-67 index, close/positive margins, and estrogen receptor (ER) negativity are predictive factors for poor OS after IBTR [16–18]. Fisher et al. also found a significant association between IBTR after BCS and OS based on the results of 15 years of follow-up of the NSABP B-06 trial, but they pointed that IBTR represents a marker rather than a cause of distant disease [19].

In the current study, we reviewed the medical records of 322 consecutive patients with stage 0–IIIA breast cancer who underwent BCS in a single center with a median follow-up period of 57 months. The first aim of this study was to identify the rates of IBTR after BCS in DCIS and invasive breast cancer and to evaluate the differences in the predictive factors for IBTR between DCIS and invasive breast cancer after BCS. The second aim of this study was to evaluate the association of IBTR and OS and distant metastasis in this study population.

## Methods

### Study patients

We retrospectively reviewed the records of 322 consecutive patients with stage 0 to IIIA breast cancer who underwent BCS at the Department of Surgery, Chungbuk National University Hospital, Cheongju, South Korea, from January 2004 to December 2010. Bilateral tumors were observed in two patients, and 324 invasive and in situ breast cancers were treated with BCS. We excluded patients who received primary chemotherapy. Because we treated patients with lobular carcinoma in situ (LCIS) as closed surveillance after pathologic confirm by excision or core needle biopsy only, the patients with LCIS were also excluded. We reviewed the medical records of each patient for clinical data including age at diagnosis, follow-up status, and information regarding outcome. Pathological parameters were evaluated, including tumor size, lymph node (LN) metastasis, estrogen receptor (ER) status, progesterone receptor (PR) status, nuclear grade, HER 2 and Ki-67 status, and results of BCS including resection margins and presence of intraductal component. The breast cancer stage was classified according to the American Joint Committee on Cancer (AJCC) TNM criteria (7th edition).

The surgical procedure consisted of a quadrantectomy or lumpectomy in all patients and axillary nodal staging with sentinel LN procedure or axillary dissection for invasive cancers. Axillary LN dissection included level 1 and 2 axillary LNs. For all cases, intraoperative frozen section diagnoses of surgical margins in eight directions were performed. When the margins were tumor-positive on frozen biopsy, additional resections were performed until the margins were shown to be negative. Surgically resected specimens were cut into tissue blocks and processed to provide permanent formalin-fixed paraffin-embedded sections. Patients with surgical margins were found to be positive on the permanent sections, most patients underwent additional re-excision surgery and the surgical margins turned out to be negative. Resection margins were classified to positive (cancer cells on inked margin), close (≤1 mm from the margin), and negative (>1 mm from the margin).

ER and PR status were determined by immunohistochemistry, and tumors with 1 % or more positively stained tumor cells were classified as positive. Any ER- or PR-positive tumors were considered as hormone receptor (HR) positive. HR negativity was defined as both ER-negative and PR-negative. HER2 status was considered positive if immunohistochemistry was 3+ or fluorescence in situ hybridization (HER-2/neu to chromosome 17 ratio) was >2.0. Proliferation activity was assessed by immunostaining with the Ki-67 antibody (Dako, Carpinteria, CA, USA). Ki67 expression was scored as the percentage of positive invasive tumor cells with any nuclear staining and recorded as mean percentage of positive cells. The Ki-67 index was evaluated by one pathologist and the proportion of proliferating cells was determined by counting at least 500 tumor cells. Ki67 index ≥14 % was considered as positive.

Hormonal therapy was performed in 236 patients (73.3 %). Tamoxifen was the most commonly used agent (136 patients, 57.8 %), followed by aromatase inhibitors (100 patients, 42.2 %). Adjuvant chemotherapy was performed according to the National Comprehensive Cancer Network (NCCN) consensus available at the time of treatment. The majority of patients (96.3 %) received adjuvant radiotherapy. Radiation therapy was administered with a total dose of 5040 cGy to the whole breast (28 × 180 cGy). A boost dose with a total of 900 cGy (5 × 180 cGy) was applied to the tumor bed. Radiation therapy to the whole breast and boost doses were administered using photons.

An IBTR was defined as a recurrent in situ or invasive carcinoma that occurred after BCS in either the skin or parenchyma of the ipsilateral breast without clinical-radiologic evidence of regional or distant disease. Distant failure was defined as all cancers that occurred at sites other than ipsilateral or contralateral breast or regional sites. The OS end-point included all deaths.

### Statistical analysis

Statistical analyses were performed using the SPSS software statistical package (Version 12 for Windows, SPSS Inc., Chicago, IL, USA). Rates of IBTR, distant failure, and OS were calculated using the Kaplan-Meier method. The significance of differences of the curves for numerous risk factors was determined using the log-rank test. Logistic regression was used to evaluate the association between covariates of interest and the probability of IBTR. Multivariate analyses were performed using Cox proportional hazards model. Cox proportional hazards model was used for univariate and multivariate analyses to determine the hazard ratios and significance of potential risk factors for OS and distant metastasis-free survival. Statistical significance was defined as a *p* value <0.05.

## Results

The median age of the study population at the time of diagnosis was 51 years (range, 23 to 79). Details on the distribution of the clinicopathological factors in the study cohort are displayed in Table 1. Fifty patients (15.5 %) had stage 0 disease, 233 patients (72.4 %) had stage I to IIA disease, and 39 patients (12.1 %) had stage IIB to IIIA disease. Multiple lesions were identified in 29 patients (9.0 %). At first operation, 13 patients were revealed as having positive margin. Of those, seven patients refused reoperation and six patients underwent re-excision; the patients who underwent re-excision were classified as having negative resection margins. Patients with having close resection margin after first operation did not undergo reoperation. Subsequently, seven patients (2.2 %, four patients with DCIS and three with invasive cancer) were classified as having positive resection margins, and five patients (1.6 %) were identified as having close resection margins of less than 1 mm. Radiation therapy was performed in 310 patients (96.3 %), and hormonal therapy was performed in 236 patients (73.3 %). Chemotherapy was administered to 198 patients (61.5 %).

**Table 1:** Patient, tumor, and treatment characteristics of 322 patients

Characteristics	*N* (%)
Age, years	
Median, range	51 (23~79)
T stage	
Tis	50 (15.5 %)
T1 (≤2 cm)	189 (58.7 %)
T2 (>2 and ≤5 cm)	83 (25.8 %)
N stage	
N0	239 (78.1 %)
N1	55 (18.0 %)
N2	12 (3.9 %)
AJCC stage	
0	50 (15.5 %)
I–IIA	233 (72.4 %)
IIB–IIIA	39 (12.1 %)
Histology	
Ductal in situ	50 (15.5 %)
Invasive ductal	244 (75.8 %)
Invasive lobular	7 (2.2 %)
Others	21 (6.5 %)
Hormonal receptor	
Positive	238 (82.1 %)
Negative	49 (16.9 %)
HER2	
Negative	235 (73.0 %)
Positive	40 (12.4 %)
Ki-67	
Negative	139 (43.2 %)
Positive	173 (53.7 %)
Nuclear grade	
Low	38 (11.8 %)
Intermediate	170 (52.8 %)
High	114 (35.4 %)
Resection margin status	
Negative	310 (96.3 %)
Close	5 (1.6 %)
Positive	7 (2.2 %)
Hormonal therapy	
Done	236 (73.3 %)
Not done	86 (26.7 %)
Chemotherapy	
Done	198 (61.5 %)
Not done	124 (38.5 %)
Radiotherapy	
Done	310 (96.3 %)
Not done	12 (3.7 %)

The median follow-up period was 57 months (range 12–133 months). Overall, 19 (5.9 %) out of 322 patients had developed IBTR. Five (10 %) out of 50 patients with DCIS and 14 (5.1 %) out of 272 patients with invasive cancer had developed IBTR. The 5-year IBTR-free survival of patients with DCIS and invasive cancer was 90.1 and 94.8 %, respectively. Contralateral recurrences were identified in six out of 272 invasive cancer patients and regional recurrence involving ipsilateral axilla and supraclavicular LN were identified in 4 out of 272 invasive cancer patients.

Tables 2 and 3 showed the predictive factors for IBTR in DCIS and invasive cancer, respectively. By univariate analysis, positive resection margin status and ER and PR negativity were associated with IBTR in DCIS patients. In contrast, ER and PR negativity, omission of radiotherapy, and high Ki67 score were associated with IBTR in invasive cancer patients. Age less than 40 years showed marginally significant predictive findings for IBTR in invasive cancer patients. By multivariate analysis, positive resection margin status (*p* = 0.007) and ER negativity (*p* = 0.03) in DCIS and omission of radiotherapy (*p* < 0.001) and ER and PR negativity (*p* = 0.001, *p* = 0.002) and high Ki-67 score (*p* = 0.04) in invasive cancer remained as significant factors predicting IBTR. Positive resection margin status showed no significant association with IBTR in invasive cancer patients.

**Table 2:** Univariate and multivariate analyses of predictive risk factors for IBTR in DCIS (*n* = 50)

Variables	Univariate		Multivariate	
	OR (95 % CI)	*p* value	HR (95 % CI)	*p* value
Age <40 years	2.0 (0.2–21.6)	0.56	1.9 (0.2–17.8)	0.54
Tumor size >2 cm	1.1 (0.6–4.2)	0.23	1.2 (0.1–5.3)	0.29
Positive margin status	4.7 (0.5–14.2)	0.02*	15.6 (2.7–66.3)	0.007*
Multiplicity	2.5 (0.4–4.4)	0.44	2.5 (0.2–2.5)	0.42
High nuclear grade	2 (0.1–9.1)	0.63	1.2 (0.1–11.5)	0.67
EIC positive	1	0.99	1	0.99
Negative ER	9.0 (1.2–65.6)	0.03*	6.7 (1.2–40.5)	0.03*
Negative PR	10.5 (1.1–105.0)	0.04*	8.9 (0.9–79.4)	0.05
Positive HER2	1	0.99	1	0.34
Comedo necrosis	2.5 (0.1–16.3)	0.40	2.2 (0.4–13.6)	0.36
Omission of radiotherapy	3.6 (0.5–25.8)	0.19	3.4 (0.6–20.1)	0.18

*p < 0.05

**Table 3:** Univariate and multivariate analyses of predictive risk factors for IBTR in invasive cancer (*n* = 272)

Variables	Univariate		Multivariate	
	OR (95 % CI)	*p* value	HR (95 % CI)	*p* value
Age <40 years	1.3 (0.8–6.7)	0.06	2.9 (0.9–9.2)	0.07
Tumor size >2 cm	1.1 (0.5–2.1)	0.85	0.9 (0.5–1.9)	0.94
Positive margin status	1	0.99	1	0.99
Multiplicity	0.78 (0.1–6.3)	0.82	1.2 (0.2–9.8)	0.81
High nuclear grade	1	0.99	2.5 (0.8–7.6)	0.86
EIC positive	1.5 (0.3–6.9)	0.59	1.4 (0.3–6.7)	0.08
Negative ER	7.9 (2.4–26.1)	0.001*	7.5 (2.3–23.8)	0.001*
Negative PR	8.2 (2.2–30.0)	0.002*	7.7 (2.2–27.8)	0.002*
Positive HER2	2.0 (0.3–16.1)	0.50	1.1 (0.7–17.3)	0.95
Positive LVI	1.4 (0.2–10.8)	0.77	1.3 (0.3–11.2)	0.72
Positive Ki-67	4.8 (1.1–21.7)	0.04*	5.0 (1.0–20.8)	0.04*
Omission of Radiotherapy	5.6 (0.3–17.2)	0.03*	50.0 (12.8–194.5)	<0.001*

*p < 0.05

At the time of analysis, all patients with DCIS were alive without distant metastasis. Out of 272 invasive cancer patients, 17 patients (6.3 %) were suffering from distant metastasis and 8 patients (2.9 %) had died. The 5-year OS and distant metastasis-free survival of 272 invasive cancer patients were 98.7 and 95.6 %, respectively.

Table 4 showed the course of disease of the patients with IBTR. The treatment of IBTR was mastectomy in all patients. Although three out of five IBTRs in DCIS patients were invasive recurrences, all patients with DCIS were alive without distant metastasis. Rather, all 13 invasive cancer patients who suffered IBTR experienced invasive IBTR. Among 272 invasive breast cancer, five (38.5 %) out of 13 patients who suffered IBTR developed distant metastasis and 3 (23.1 %) had died. The 5-year OS of patients with IBTR was inferior to those without (84 vs. 98 %). And, the 5-year distant metastasis-free survival of patients with IBTR was inferior to those without (63.3 vs. 96.5 %). However, the differences in outcome did not reach statistical significance.

**Table 4:** Analyses of 19 patients who had experienced IBTR

No.	Age	Histology of primary tumor	Subtype	RM	RT	Interval to IBTR (months)	Histology of IBTR	2nd recurrence	Interval to 2nd recurrences (mo.)	F/U duration (months)	Overall survival (months)
1	60	DCIS	ER +	Neg.	Not done	12	DCIS	none	N/A	41	80
2	26	DCIS	ER -	Pos.	Done	13	DCIS	none	N/A	126	128
3	52	DCIS	ER +	Pos.	Done	14	IDC	none	N/A	26	30
4	56	DCIS	ER -	Neg.	Not done	20	IDC	none	N/A	42	114
5	43	DCIS	ER -	Neg.	Done	47	IDC	none	N/A	52	52
6	31	Metaplastic	TN	Neg.	Done	14	Metaplastic, TN	none	N/A	30	30
7	45	IDC	TN	Neg.	Done	8	IDC, TN	lung	6	18	18 (dead)
8	48	IDC	TN	Neg.	Not done	14	IDC, TN		N/A	49	74
9	44	IDC	TN	Neg.	Done	15	IDC, TN		N/A	34	34
10	49	IDC	TN	Neg.	Done	16	IDC, TN	lung	9	35	35 (dead)
11	33	IDC	HER2	Neg.	Done	17	IDC, TN		N/A	62	69
12	36	IDC	Luminal	Neg.	Done	21	IDC, Luminal	bone	10	36	36
13	41	IDC	TN	Neg.	Done	22	IDC, HER-2	lung	15	38	42
14	65	IDC	Luminal	Neg.	Not done	30	IDC, Luminal		N/A	36	36
15	43	IDC	Luminal	Pos.	Not done	30	IDC, Luminal		N/A	38	47
16	44	IDC	TN	Neg.	Done	34	IDC, TN		N/A	52	52
17	51	IDC	Luminal	Neg.	Done	39	IDC, Luminal		N/A	49	60
18	48	IDC	TN	Neg.	Done	47	IDC, TN		N/A	63	77
19	39	IDC	HER2	Neg.	Done	52	IDC, HER2	lung	5	78	78 (dead)

*Neg.* negative, *Pos.* positive, *TN* triple negative

The univariate and multivariate analyses of potential prognostic factors for OS and distant metastasis-free survival in invasive cancer patients are shown in Tables 5 and 6. LN positivity, advanced stage, and experience of IBTR were associated with poor OS and distant metastasis on univariate analysis. LN positivity (*p* = 0.03) and experience of IBTR (*p* < 0.001) remained as highly significant in multivariate analysis for predicting OS and distant metastasis-free survival.

**Table 5:** Univariate and multivariate analyses of potential risk factors for overall survival in patients with invasive cancer (*n* = 272)

Variables	Univariate		Multivariate	
	HR (95 % CI)	*p* value	HR (95 % CI)	*p* value
Age <40 years	0.6 (0.2–2.4)	0.51	1.2 (1.5–9.7)	0.88
T stage, T2 (vs. T1)	1.8 (0.9–3.2)	0.12	2.1 (0.3–5.7)	0.54
Positive LN	3.6 (1.5–11.3)	0.02*	6.9 (1.2–38.4)	0.03*
Stage, IIB-IIIA	4.8 (1.2–19.6)	0.03*	4.9 (1.6–29.3)	0.02*
IBTR	21.9 (4.7–101.9)	<0.001*	31.8 (6.1–165.3)	<0.001*
Positive margin status	1	0.85	1	0.93
Omission of Radiotherapy	1	0.78	1	0.92
Negative ER	1.6 (0.5–4.4)	0.40	3.1 (0.7–12.3)	0.12
Negative PR	1.8 (0.7–4.9)	0.26	3.4 (0.8–14.3)	0.09
Positive HER2	1.2 (0.1–8.3)	0.92	1	0.95
Positive LVI	1.2 (0.2–1.3)	0.84	2.3 (0.3–18.8)	0.43
Positive Ki-67	1.1 (0.4–2.9)	0.87	1.5 (0.3–5.9)	0.64
High histologic grade	1	0.84	1	0.97

*p < 0.05

**Table 6:** Univariate and multivariate analyses of potential risk factors for distant metastasis in patients with invasive cancer (*n* = 272)

Variables	Univariate		Multivariate	
	HR (95 % CI)	*p* value	HR (95 % CI)	*p* value
Age <40 years	1.5 (0.4–5.7)	0.51	1.2 (0.3–4.2)	0.78
T stage, T2 (vs. T1)	1	0.83	1	0.98
Positive LN	4.8 (1.3–17.7)	0.02*	3.4 (0.9–12.3)	0.04*
Stage, IIB–IIIA	3.1 (1.1–8.4)	0.03*	3.83 (1.4–10.8)	0.07
IBTR	11.5 (3.9–33.4)	<0.001*	14.4 (4.8–43.1)	<0.001*
Positive margin status	1	0.99	1	0.95
Omission of Radiotherapy	1	0.99	1	0.90
Negative ER	1.6 (0.5–4.4)	0.39	2.7 (0.1–10.6)	0.25
Negative PR	1.8 (0.6–4.9)	0.22	1.8 (0.7–4.8)	0.90
Positive HER2	1.2 (0.2–8.4)	0.92	1	0.98
Positive LVI	1.2 (1.0–10.1)	0.84	1	0.99
Positive Ki-67	1.1 (0.4–2.9)	0.87	1.2 (0.4–3.0)	0.65
High histologic grade	1	0.833	1	0.98

*p < 0.05

## Discussion

The purpose of this study was to evaluate the differences in the rates and predictive risk factors for IBTR between DCIS and invasive breast cancer, from 322 consecutive patients with primary breast cancer undergoing BCS at a single center. We also analyzed their prognostic influence of IBTR on patients’ overall survival and distant metastasis.

In the current study, 19 (5.9 %) patients, 5 (10 %) out of 50 with DCIS and 14 (5.1 %) out of 272 with invasive cancer, had developed IBTR. These results are comparable to those of previous studies regarding IBTR after BCS [1, 2, 7]. In those studies, the incidence of IBTR after BCS for invasive cancer was found to vary between 5 and 10 % over 5 years, and the risk continues over a longer period of follow-up. However, our results showed some high rates of IBTR in comparison to a previous study using a Korean database, which reported an the overall 5-year rate of IBTR as low as 1.6 % in invasive cancer [4]. In that study, patients who did not receive adjuvant radiotherapy and those with positive surgical margins were excluded. Because we analyzed consecutive breast cancer patients who underwent BCS in a single center, five patients with positive/close margins and three patients who did not undergo radiotherapy were included. We therefore assume these factors to be the reason for our higher IBTR rate. The 5-year rate of IBTR after BCS in DCIS has been reported to be about 5–10 % [8, 9, 20]. Our study showed comparable findings, with a rate of IBTR after DCIS of 10 % with a median follow-up period of 57 months.

In this study, we wanted to know whether or not there was a difference, in predictive factors for IBTR between DCIS and invasive cancer. In patients with DCIS, multivariate analysis revealed that positive margin status and ER negativity remained as independent predictors for IBTR. In contrast, omission of radiotherapy, HR negativity, and high Ki67 score were the independent predictive factors for IBTR in invasive cancer. Although we had a small study population, multiplicity, young age, and extensive intraductal component (EIC), LVI, and HER-2 positivity showed little or no clinical significance as predictive factors for IBTR in this study. Larger studies with longer follow-up are needed to understand more detailed predictive factors for IBTR.

However, in this study, HR negativity was the strong independent predictive factor for IBTR in both DCIS and invasive cancer. This is a consistent finding with others who showed patients with triple-negative breast cancers behave less favorably than luminal breast cancers and almost as poorly as HER2/neu over-expressing cancers in terms of locoregional recurrence following BCS [3]. Demicheli et al. analyzed recurrence peak after BCS according to ER status and showed IBTR risk peak delay observed in ER-positive tumors than ER-negative tumors after BCS. They suggest that the microenvironment within the residual breast tissue may enforce more stringent constraints upon ER-positive breast tumor cell growth than other tissues, prolonging the latency of IBTR. This local environment is, however, apparently less constraining to ER-negative cells [21]. Additionally, there were many studies about the effect of subtype of IBTR on prognosis. Ishitobi et al. [18] showed that IBTR with luminal A subtype showed the most favorable outcome in terms of distant recurrence than others and breast cancer subtype was an independent predictor associated with distant recurrence after IBTR. In other study, locoregional failure showed significantly larger effect on mortality and distant disease in ER-negative patients than in ER-positive patients [17]. Aggressive local control and adjuvant therapy should be considered for patients with HR-negative breast cancer who undergo BCS.

In our study population, all three invasive breast cancer patients who did not undergo radiotherapy developed IBTR. Despite our small study population and the small number of events, these results are consistent with the findings of NSABP B-06, which showed that patients treated with radiation remained with low IBTR as compared to those receiving no radiation through 9 years of follow-up, regardless of age, nodal status, and tumor size [22]. Radiotherapy should be performed in all patients of invasive cancer who treated with BCS.

Likewise, postoperative radiotherapy after BCS has known as the standard treatment in DCIS patients. However, there remains variation in the use of radiotherapy in favorable type of DCIS. In a series published by Silverstein et al., patients with small lesions, with favorable histologies, and of low to intermediate grade with widely negative margins (>1 cm) treated by BCS alone reported an IBTR rate as low as 6 % at 5 years [23]. Rudloff et al. [24] reported that radiotherapy reduced IBTR only in cases of close margins below 1 cm and the effect of radiotherapy on IBTR risk was influenced by the margin width and the volume of disease near the margin. Kim et al. [9] analyzed the outcome of local excision alone in patients with small size (<1 cm) DCIS and reported that radiotherapy is necessary in patients with resections margin of <1.0 cm. But in recent prospective studies, in good-risk subset of patients with DCIS, with a median follow-up of 7 years, the local failure rate was decreased significantly with the addition of RT [25]. In our study, two out of nine patients with DCIS who omitted radiotherapy developed IBTR. This was the similar finding with recent studies, and careful selection should be taken for radiotherapy omission in patients with DCIS after BCS.

Although there were three invasive cancer patients with positive margins after BCS, marginal status had not reach statistical significance in terms of predicting IBTR in patients with invasive cancer. This is probably due to the limited number of patients and events with short follow-up period. Recent ASCO guidelines showed the use of “no ink on tumor” should be the standard for an adequate margin in invasive cancer. A positive margin, defined as ink on invasive cancer or DCIS, is associated with at least a twofold increase in IBTR; this increased risk in IBTR is not nullified by delivery of a boost, delivery of systemic therapy, or favorable biology [12]. Further detailed comparison of close and positive margin after BCS in invasive cancer patients should be needed in further larger study. In contrast, although there were small number of events and patients with DCIS, positive marginal status was an independent predictive factor for IBTR in DCIS. Through meta-analysis, Wang et al. [11] reported that negative margins should be obtained for DCIS patients regardless of radiotherapy, and a negative margin threshold greater than 10 mm is the best option compared with other margin thresholds. According to the 2015 national comprehensive cancer network (NCCN) guideline, in patients with DCIS, margins greater than 10 mm are widely accepted as negative and margins less than 1 mm are considered inadequate. There should be more careful attention to achieve negative margin in patients with DCIS who underwent BCS.

Probably due to the limited number of patients and the short follow-up period in our study, we could not demonstrate statistically significant differences on survival curve according to IBTR. But, among 272 invasive cancer, the 5-year OS of patients with IBTR was inferior to those without (84 vs. 98 %). And, the 5-year distant metastasis-free survival of patients with IBTR was inferior to those without (63.3 vs. 96.5 %). On univariate and multivariate analyses, LN metastasis and IBTR revealed as independent predictive factor for OS and distant metastasis-free survival in invasive cancer. Many investigators have reported that IBTR, especially early IBTR after BCS, is associated with subsequent mortality. In addition, clinicopathologic features of the recurrent tumor including LVI, high-grade histology, high Ki-67 index, close/positive margins, and ER negativity are known as predictive factors of poor OS after IBTR [16–18]. Investigations on larger study populations with longer follow-up periods should be conducted to assess the more detailed clinical impact of IBTR on overall patient outcomes. This information can be helpful in optimizing local and systemic therapy at the time of original diagnosis for patients undergoing conservative treatment breast cancer. When we treat patient with BCS, identification of individual patient’s clinical and pathologic factors that predict for IBTR and efforts to lowering IBTR should be needed.

Our analysis is a single institution retrospective study of small number of patients with a relatively short median follow-up of 57 months. A longer follow-up period is needed to estimate the long-term outcome. In addition, selection bias is expected due to the heterogeneities of the study population in the clinical and pathological characteristics and treatment methods.

## Conclusions

The ER and PR negativity was revealed as an independent predictive factor for IBTR after BCS in both DCIS and invasive breast cancer. Meanwhile, positive margin status in DCIS and the omission of radiotherapy in invasive cancer, respectively, were the independent prognostic factors for IBTR. LN metastasis and experience of IBTR were found to be associated with poor overall survival and distant metastasis in invasive cancer patients after BCS. More efforts should be made to reduce IBTR when treating patient with BCS. And, aggressive local control and adjuvant therapy should be considered in HR-negative patients with advanced stage who receive treatment with BCS.

### Ethics approval and consent to participate

The protocol for the study was approved by the Institutional Review Board (approval number, 201410012) of the Chungbuk National University College of Medicine, and the study was carried out in agreement with the applicable laws and regulations, good clinical practices, and ethical principles as described in the Declaration of Helsinki.

## Abbreviations

DCIS: ductal carcinoma in situ
EIC: extensive intraductal component
EIC: extensive intraductal component
ER: estrogen receptor
HER2: human epidermal growth receptor 2
HR: hormonal receptor
IBTR: ipsilateral breast tumor recurrence
IDC: invasive ductal carcinoma
LVI: lymphovascular invasion
PR: progesterone receptor
RM: resection margin
RT: radiotherapy
